# Perceived use and value of reproductive, problem-solving, and athlete-initiated teaching by coaches and athletes

**DOI:** 10.3389/fpsyg.2023.1167412

**Published:** 2023-05-24

**Authors:** Koray Kılıç, Mustafa Levent Ince

**Affiliations:** ^1^Faculty of Sport Sciences, Kırşehir Ahi Evran University, Kırşehir, Türkiye; ^2^Physical Education and Sports Department, Faculty of Education, Middle East Technical University, Ankara, Türkiye

**Keywords:** coaching, teaching methods, reproductive, problem-solving, athlete-initiated

## Abstract

In the sports coaching environment, it is recognized that developing athletes’ autonomy and problem-solving skills are crucial to support holistic development and ensure optimal performance. However, there needs to be more information on how coaches use and value different teaching methods in training and how athletes perceive and value these methods. This study aimed to examine coaches’ and athletes’ perceptions of the use and value of reproductive, productive problem-solving, and productive athlete-initiated teaching methods. To this end, the Coaches’ Use of Teaching Methods Scale which is validated for the use of coaches and athletes, was applied to 70 coaches and their 294 athletes of youth sports teams purposefully selected from four cities in Türkiye. Data were analyzed by nonparametric methods, including Friedman’s and Mann–Whitney tests (*p* < 0.05). Although there were statistically significant differences between the responses of coaches and athletes regarding the use of different teaching methods in their training and the value they gave to these methods, both groups marked the frequent use of reproductive, occasional use of productive problem-solving and rare use of productive athlete-initiated teaching methods during training. The value given to productive athlete-initiated teaching methods in terms of enjoyment, learning, and motivation by the athletes was higher than the value given to them by the coaches. The study’s findings strongly indicate the coaches’ professional needs in their pedagogical knowledge, specifically on their value perceptions of productive problem-solving and productive athlete-initiated teaching methods and the capacity to apply them.

## 1. Introduction

The primary indicator of effective coaching is athletes’ learning and its positive effect on their development in sport (Côté and Gilbert, 2009). Prominent athlete development models such as Long-Term Athlete Development (Balyi et al., 2013) and the Developmental Model of Sport Participation (the DMSP; Côté, 1999; Côté et al., 2007) emphasize biological age and maturation-dependent sport-specific competence (e.g., technique and fitness), and psychosocial skills such as athletes’ confidence and character development. These developmental frameworks provide coaches with conceptual guidance for coaching aims, content identification, implementation, and measurement and evaluation of their practice outcomes to improve athletes’ learning and performance at a sustainable level (e.g., Lauer and Dieffenbach, 2013).

Based on the coaching aims, setting the objectives, identifying the content, implementing training, and measuring and evaluating the outcomes should be well aligned to ensure athletes’ optimum development (Bompa and Haff, 2009; Lara-Bercial et al., 2013). Studies examining the aims of contemporary coaching and the appropriate content to reach those aims stress the holistic development of athletes in all developmental areas and using relevant content during the training (sport-specific technique, fitness, tactics, psychosocial, and emotional development; Fraser-Thomas et al., 2005; Côté et al., 2010). In coaching and teaching literature, as the athletes’ performance develops, a need for higher-order learning in all developmental aspects of athletes with the recognition of their individual differences is underlined (e.g., Wikeley and Bullock, 2006; Cushion and Nelson, 2013). Specifically, athletes should develop deep learning capacity by having more opportunities for application, synthesis, evaluation, and creation in the cognitive domain (Thorpe, 1997; Potrac and Cassidy, 2006; Cassidy et al., 2008), adaptation and organization in the psychomotor domain, and characterization by a value or value complex in the affective domain. Athletes’ superficial learning in these developmental areas may not be sufficient to realize the current training aims and improve sports performance significantly (Wikeley and Bullock, 2006, p. 18).

Presenting the content (how to teach/instruct) is a critical dimension of coaching knowledge (Gilbert and Côté, 2013), which enables coaches’ training implementation. Coaches’ instructions may be beneficial or harmful to athletes’ development depending on the extent that they are informed about the optimal strategies for athletes’ learning and performance needs (Gearity, 2012). Previous work on instructional aspects of coaching naturally depends on the theories of learning and instruction (e.g., Kidman, 2005; Armour, 2010). Accordingly, the intended learning in sport occurs most effectively when the learning intention and instructional strategies or teaching methods are correctly matched. Coaches need to be aware of the consequences of their instructional practices for coaching effectiveness (e.g., Cassidy et al., 2008, p. 33).

For this reason, it is essential for coaches to understand the theories of learning (how athletes learn) and instruction (how coaches should teach) and to use them effectively in practice (Light, 2008; Roberts and Potrac, 2014). Whenever the coaching focuses on ensuring higher-order learning, independent of coaching context (Cassidy et al., 2008), direct teaching methods (reproductive: replication of a known model) would not be sufficient; coaches should also use productive teaching methods (production of knowledge and skills new to the athlete and/or coach), including productive problem-solving and productive athlete-initiated approaches in their training to facilitate deep learning (Wikeley and Bullock, 2006; Ertmer and Newby, 2013; Pill et al., 2021). That brings about the necessity of coaches’ regular assessment of athletes’ higher-order learning in cognitive, psychomotor, and affective domains due to coaching practices. The current study focuses on the perceived use of various instructional strategies and teaching methods during training by coaches and athletes. The following paragraphs in this section will synthesize the related literature to create a rationale for the study.

In the sports education literature, teaching methods are usually characterized by Mosston and Ashworth’s (2008) spectrum of teaching styles. Mosston and Ashworth’s Spectrum of Teaching Styles identify 11 different teaching methods in a continuum from teacher-centered to learner-centered: A: Command, B: Practice, C: Reciprocal, D: Self-Check, E: Inclusion, F: Guided Discovery, G: Convergent Discovery, H: Divergent Discovery, I: Learner Designed Individual Program, J: Athlete-initiated, and K: Self Teaching. Teachers’ dominance of decision-making processes in planning, implementation, and measurement and evaluation of an outcome in the teaching process decreases as teaching styles progress from A to K. Styles from A to E use a direct teaching approach. Therefore, they are called reproductive methods of teaching in the sports education setting. The styles from E to K are more learner-centered, and the learner’s discovery of new information or skills is at their core. Due to their focus on learners’ discovery by problem-solving, styles from E to H can be named productive problem-solving (henceforth problem-solving) teaching methods. On the other hand, learner autonomy in the styles from I to K is dominant in planning, implementation, and measurement and evaluation of the outcome. They can be named under the title of productive athlete-initiated (henceforth athlete-initiated) teaching methods (Kilic and Ince, 2017; Pill et al., 2021).

There is limited research on coaches’ use of teaching methods in their practices, although in many sport pedagogy textbooks (e.g., Kidman, 2005; Jones, 2006; Cassidy et al., 2008; Mitchell et al., 2020) and relevant work in teaching (e.g., Light, 2008) the importance of teaching methods, especially the use of learner/athlete-centered methods, is highlighted by well-known scholars in the field. Ample research indicates the use and value given to the various teaching methods by physical education teachers in school physical education classes. Those studies in physical education settings reported heavy use of teacher-centered reproductive teaching methods (e.g., command and practice) but occasional use of learner-centered approaches (e.g., problem-solving and athlete-initiated methods; Curtner-Smith et al., 2001; Cothran et al., 2005; Ince and Hunuk, 2010). Interestingly, these studies also reported that physical education teachers highly valued reproductive methods while valuing the learner-initiated teaching methods for learners’ learning, motivation, and enjoyment less.

A few pilot studies have been conducted to assess coaches’ use of teaching methods so far (Ince and Kilic, 2016; Kilic and Ince, 2017). The findings of this work were similar to that of the school physical education setting. The preliminary results indicated the coaches’ tendency to use reproductive methods, especially command and practice, while rarely using problem-solving and athlete-initiated teaching methods.

Studies examining instruction in physical education and coach development settings have mainly focused on teachers’ practices (e.g., Ince and Hunuk, 2010) and coaches (Potrac and Jones, 2009; Trudel et al., 2010). While the research on examining coaching behaviors indicated instruction is the most preferred and motivating form of coaching practice by athletes (Gearity, 2012) coaches may lack the knowledge and ability to apply the types of instruction for different learning situations. Consequently, their instruction can be poor in meeting youth athletes’ unique learning needs (Gearity, 2012). There needs to be a more profound understanding of coaches’ instructional practices and tendencies and, more importantly, athletes’ perceptions of coaching practices to improve coaching effectiveness. To our knowledge, how the athletes perceive the specific teaching methods coaches apply needs to be clarified in the coaching literature. Exploring the use of and value given to reproductive, problem-solving, and athlete-initiated teaching methods in coaching settings could provide critical information on the use and preference of athlete-centered discovery strategies in sports education settings better with the view of both coaches and their athletes. Such information would help design professional development programs for the coaches’ professional knowledge of improving youth athletes’ higher-order learning in the defined developmental areas.

Considering the rationale mentioned above, this study explores the coaches’ and their athletes’ perceived use and value of reproductive, problem-solving, and athlete-initiated teaching methods. To this end, the following research questions were asked;

1. What are the perceptions of coaches and athletes about the use of reproductive, problem-solving, and athlete-initiated teaching methods during training?

- Are there any significant differences between the coaches’ perceived use of reproductive, problem-solving, and athlete-initiated teaching methods during the training?
- Are there any significant differences in coaches’ use of reproductive, problem-solving, and athlete-initiated teaching methods during the training by the athletes’ perception?

2. Are there any significant differences between coaches and athletes in value given to reproductive, productive problem-solving, and productive athlete-initiated teaching methods concerning enjoyment, learning, and motivation during training?

## 2. Materials and methods

### 2.1. Participants

Coaches (*n* = 70) and their athletes (*n* = 294) of youth sports teams from Ankara, Istanbul, Bartın, and Kırşehir cities of Türkiye participated in the study. The coaches were between 18 and 50 years of age (*M* = 32.69), representing each level of coaching certificate in a 5-level coaching system (*M* = 2.61). In Türkiye, the first and second levels of coaching are equal to developmental level certification, while the higher levels represent elite coaching contexts at national and international levels (GSD, 2019). The athletes’ ages were between 12 and 18, with an average of 4.90 years of sport experience (SD = 2.52). The athletes train 4.88 days a week (SD = 1.33). With respect to gender representation, 9 and 61 out of 70 coaches were women and men, respectively. Out of 294 athletes, 130 of them were women, and 164 of them were men. The representation of low women coaches in the sampling is related to the relatively low representation of women coaches in the study setting. According to Koca (2018, p.142), 20–25% of the coach population in Türkiye are women. Demographics of coaches and athletes based on sports type are presented in Table 1.

**Table 1:** Demographic characteristics of participants.

Sport		Coach		Athlete
	*n*	Mean age (SD)	*n*	Mean age (SD)
Badminton	9	32.78 (8.32)	44	13.66 (0.68)
Basketball	12	33.08 (9.1)	62	15.08 (1.44)
Box	2	41.50 (10.61)	14	16.36 (1.22)
Judo	2	32.00 (0.00)	8	14.25 (1.75)
Kick box	5	32.40 (5.18)	30	16.20 (1.03)
Soccer	4	35.50 (5.80)	18	14.94 (0.73)
Swimming	4	27.75 (2.1)	25	13.32 (1.03)
Taekwondo	4	29.00 (2.16)	13	14.92 (1.26)
Tennis	7	35.29 (6.10)	32	14.47 (1.19)
Athletics	10	33.50 (6.98)	25	15.64 (1.44)
Volleyball	7	31.00 (6.83)	16	14.31 (1.45)
Wrestling	4	29.75 (5.91)	7	14.43 (1.13)
Gender				
Women	9		130	
Men	61		164	
Total	70		294	

### 2.2. Data collection instruments

A multi-perspective approach was adopted to measure coaches’ use and value perceptions of teaching methods. In examining the teaching methods applied during training and to what extent these methods were valued, the two versions of the “Coaches’ Use of Teaching Methods Scale” (CUTEMS-Coach/Athlete; Kılıç and İnce, 2019, 2021a) were used. The scales for coaches and athletes share the same structure but are worded slightly differently to reflect who is rating. For both versions of the scale, the items were generated based on the adapted form of the “Use of Teaching Styles and Perceptions of Styles Questionnaire” (Kulinna and Cothran, 2003) for the Turkish physical education context by Ince and Hunuk (2010). The scale items include 11 scenarios and four questions answered for each on a 5-point Likert scale (never to always). The 11 scenarios are broken down into three approaches of teaching methods, namely reproductive (items 1–5), problem-solving (items 6–8), and athlete-initiated (items 9–11) for both of the scales (See Table 2 for sample scenarios). The first question of each scenario is to determine the level of ‘coaches’ use of a teaching method (I train my athletes with this method/My coach trains with this method), and the other three questions are to examine the value perceptions about the teaching method regarding “enjoyment’, “learning’, and “‘motivation’ (e.g., I think this method will make training fun).

**Table 2:** Sample scenarios for reproductive, problem-solving, and athlete-initiated teaching.

Scenario samples
*Reproductive*
1. The coach breaks down the skills into parts and demonstrates the right way to perform the skill. Athletes try to move when and exactly how the coach tells them. The coach provides feedback and the athletes try to emulate the coach’s model.
2. The coach makes several stations in the gym where athletes work on different parts of a skill or different skills. Athletes rotate around the stations and do the tasks at their own pace. The coach moves around and helps athletes when needed.
*Productive problem-solving*
6. The coach asks athletes to discover a solution to a movement problem. The coach asks athletes a series of specific questions and the athletes try out their answers until they discover the right answer that the coach wanted them to discover.
7. Athletes try to learn a skill or concept by using logical reasoning. The coach asks a question and athletes try to reason and think about different solutions. By critically thinking about the question and trying solutions, athletes can discover the single, right answer.
*Productive athlete-initiated*
10. The athlete decides what will be learned as well as how it will be learned. The coach and athlete set some basic criteria, but the athlete is responsible for all the decisions about how and what to learn. The coach can help with information if the athlete needs it.
11. The athlete decides everything about learning something new. They even decide if they want to involve the coach or not. The coach accepts the athlete’s decisions about learning.

Scenarios are adapted from Kulinna and Cothran (2003) by Kılıç and İnce (2019).

The scales were developed by first conducting exploratory factor analysis (EFA) and confirmatory factor analysis (CFA) on two different data comprised of 275 athletes and 148 athletes from various sports, respectively (Kılıç and İnce, 2019). EFA findings revealed a 3-factor construct in line with the theoretical foundations (i.e., behaviorist, cognitivist, and constructivist approaches) the items fit in, with internal consistency values ranging from 0.72 to 0.81. CFA findings proved the construct validity of the scale subdimensions (*χ*^2^/*df* = 1.34; GFI = 0.93; CFI = 0.94; TLI = 0.92; RMSEA = 0.05). Then, the validity and reliability of the scale sub-dimensions for coaches were tested by examining the concurrent validity and internal consistency of ‘coaches’ and their ‘athletes’ ratings (Kılıç and İnce, 2021a). Findings showed strong correlations between the ratings of coaches and athletes for each subdimension (reproductive, *r* = 0.83, *p* < 0.01; problem-solving, *r* = 0.62, *p* < 0.01; athlete-initiated, *r* = 62, *p* < 0.01). Internal consistency values of the examined factors ranged from 0.69 to 0.86.

### 2.3. Data collection procedures

Before data collection, permission was obtained from the Research Ethics Committee of Middle East Technical University (No: 28620816/154). A purposeful sampling strategy was adopted to reach a highly representative number of competitive youth sports coaches and their athletes and select the coaches and athletes who have worked together for at least 1 year. While primarily aiming at collecting data within the largest cities of Türkiye (Ankara and İstanbul), two other smaller cities of Türkiye (Bartın and Kırşehir) were also included in the data collection to ensure the representativeness of coaches’ use of teaching methods in the study setting examined.

The first researcher collected the data by visiting the sport club settings. Coaches and their athletes completed the adapted versions of the scales (CUTEMS-Coach/Athlete). Athletes and coaches completed the scales separately to ensure the trustworthiness of responses. Coaches and athletes rated the scales considering the current coaching practices regarding teaching methods. Coaches and athletes completed the scales in approximately 15 min.

### 2.4. Data analysis

Data were initially screened for missing cases and matched the representation of coaches and their athletes in the data set. Fifty-six athletes from artistic gymnastics and’ ‘women’s rugby were eliminated from the data set due to a lack of their coaches’ data. Then, the data were examined to meet the normality assumptions. As coaches’ and athletes’ data on the use of teaching methods and the value given to teaching methods needed to meet the normality assumptions, the analyses were conducted by nonparametric tests. Perceived use of teaching methods data of coaches and athletes were analyzed separately by the Friedman test for the first research question.

Coaches’ and athletes’ comparison of the value given to teaching methods regarding enjoyment, learning, and motivation were analyzed by the Mann–Whitney *U* test (*p* < 0.05).

## 3. Results

Rq1. What are the perceptions of coaches and athletes about the use of reproductive, problem-solving and athlete-initiated teaching methods during training?

Regarding the first research question, according to the descriptive analysis, both coaches and athletes reported the predominant use of reproductive teaching methods during the training. Data from coaches and athletes also indicated the occasional use of problem-solving teaching methods and almost no use of athlete-initiated teaching methods during training (Table 3).

**Table 3:** Coaches’ and their athletes’ reports on the use of reproductive, problem solving, and athlete-initiated teaching methods during the training.

Teaching methods	Data source	
	Coach (*n* = 70) Mean (SD)	Athlete (*n* = 294) Mean (SD)
Reproductive^*^	4.66 (0.30)	3.70 (0.72)
Problem-solving^*^	3.15 (0.38)	2.78 (0.96)
Athlete-initiated^*^	1.45 (0.35)	1.67 (0.70)

* Significant differences among the reported use of teaching methods during the training. In a 5 level Likert Scale (1 = Never, 2 = Rarely, 3 = Sometimes, 4 = Usually; 5 = Always).

(a). Are there any significant differences between the coaches’ perceived use of reproductive, problem-solving, and athlete-initiated teaching methods during the training?

**Figure 1:**
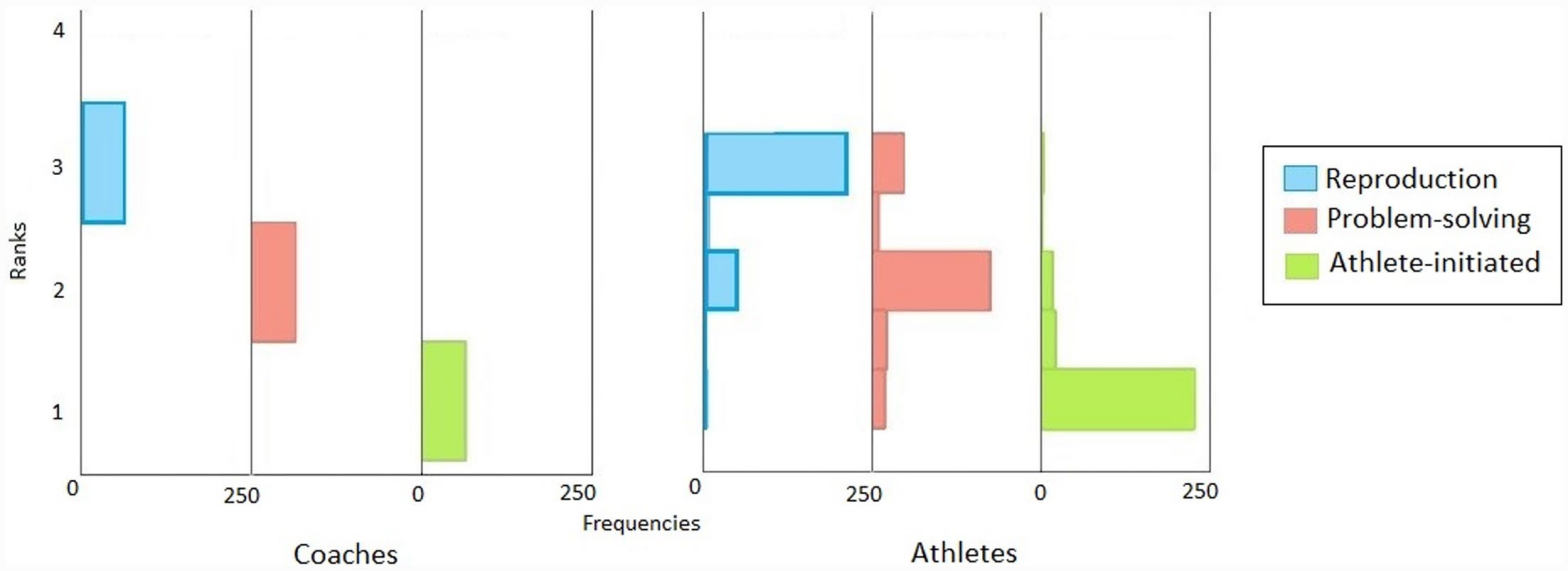
Coaches’ and their athletes’ perception on the use of reproductive, problem-solving and athlete-initiated teaching methods during training.

According to Friedman’s test, there were significant differences in the coaches’ use of reproductive, problem-solving, and athlete-initiated teaching methods during the training, *χ*^2^(2) = 140.000, *p* = 0.001. *Post hoc* analysis with a Bonferroni correction indicated that the mean rank of perceived use of reproductive, problem-solving, and athlete-initiated teaching methods were 3, 2, and 1, respectively. There were significant differences between the use of reproductive and problem-solving (*Z* = −5.916, *p* = 0.001), reproductive and athlete-initiated (*Z* = 11.832, *p* = 0.001), and problem-solving and athlete-initiated (*Z* = 5.916, *p* = 0.001) teaching methods by coaches. Coaches reported the dominant use of reproductive teaching methods and the rare use of athlete-initiated teaching methods during training (Figure 1).

(b). Are there any significant differences in coaches’ use of reproductive, problem-solving, and athlete-initiated teaching methods during the training by the athletes’ perception?

Friedman’s test showed significant differences in coaches’ use of reproduction, problem-solving, and athlete-initiated teaching methods during training, according to the athletes’ perception, *χ*^2^(2) = 410.870 *p* = 0.001. *Post hoc* analysis with a Bonferroni correction indicated that the mean rank of perceived use of reproductive, problem-solving, and athlete-initiated teaching methods were 2.78, 2.08, and 1.14, respectively. There were significant differences between the use of reproductive and problem-solving (*Z* = 8.433, *p* = 0.001), reproductive and athlete-initiated (*Z* = 19.898, *p* = 0.001), and problem-solving and athlete-initiated (*Z* = 11.465, *p* = 0.001) teaching methods used during training. The athletes reported their coaches’ dominant use of reproductive teaching methods and the rare use of athlete-initiated teaching methods during training (Figure 1).

Rq2. Are there any significant differences between the coaches and the athletes in value given to reproductive, problem-solving and athlete-initiated teaching methods concerning enjoyment, learning and motivation during training?

According to the descriptive data analysis, in terms of enjoyment, learning, and motivation, coaches’ and athletes’ values given to teaching methods from the highest to the least were reproductive, problem-solving, and athlete-initiated teaching methods, respectively (Table 4).

**Table 4:** The coaches’ and their athletes’ value perception on the use of reproductive, problem solving, and athlete-initiated teaching methods regarding enjoyment, learning, and motivation during training.

Teaching methods	Value component	Data source	
		Coach (*n* = 70) Mean (SD)	Athlete (*n* = 294) Mean (SD)
Reproductive	Enjoyment	3.69 (0.60)	3.73 (0.73)
	Learning^*^	3.92 (0.58)	4.16 (0.62)
	Motivation^*^	3.85 (0.60)	4.04 (0.68)
Problem-solving	Enjoyment	3.50 (0.90)	3.63 (1.39)
	Learning	3.80 (0.88)	3.90 (0.81)
	Motivation^*^	3.67 (0.89)	3.89 (0.89)
Athlete-initiated	Enjoyment^*^	2.45 (1.10)	3.41 (1.05)
	Learning^*^	2.48 (1.14)	3.49 (1.05)
	Motivation^*^	2.50 (1.21)	3.58 (1.04)

* Significant differences between the coaches and athletes in value given the teaching methods. In a 5 level Likert Scale (1 = Never, 2 = Rarely, 3 = Sometimes, 4 = Usually; 5 = Always).

A Mann–Whitney test indicated that athletes valued reproductive teaching methods in terms of learning (Athletes Mdn = 4.2; Coaches Mdn = 4.0), *U* = 7760.5, *p* = 0.001 and motivation (Athletes Mdn = 4.0; Coaches Mdn = 3.8), *U* = 8,230,5 *p* = 0.009, problem-solving teaching methods in terms of motivation (Athletes Mdn = 4.0; Coaches Mdn = 3.7), *U* = 8640.0, *p* = 0.035, and athlete-initiated teaching methods in terms of enjoyment (Athletes Mdn = 3.3; Coaches Mdn = 2.3), *U* = 5453.0, *p* = 0.001, learning (Athletes Mdn = 3.7; Coaches Mdn = 2.3), *U* = 5193.0, *p* = 0.001) and motivation (Athletes Mdn = 3.7; Coaches Mdn = 2.3), *U* = 5079.5, *p* = 0.001, were significantly higher than those given by the coaches. There was no significant change in the value given to the reproductive teaching methods regarding enjoyment, and problem-solving teaching methods regarding enjoyment and learning between the coaches and athletes (*p* > 0.05; Table 4).

## 4. Discussion

This study aimed to examine the coaches’ and their athletes’ perceived use and value given to reproductive, problem-solving, and athlete-initiated teaching methods during training in the competitive youth sport context.

Initial descriptive analysis of the responses of the coaches and their athletes on coaches’ use of the teaching methods indicated that the coaches widely use reproductive teaching methods while occasionally using problem-solving teaching methods. Little or no use of athlete-initiated teaching methods during training was reported. A Friedman’s test with *post hoc* analysis of the coaches’ and the athletes’ responses revealed significant differences in the use of each teaching method. The athletes perceived the use of reproductive and problem-solving teaching methods as significantly lower than the coaches did during training. While the coaches and the athletes perceive the coaches’ rare use of athlete-initiated teaching methods, the athletes scored higher than the coaches in their use.

This present study’s findings give insight into the coaches’ instructional knowledge and practices reflecting the perspective of their athletes in addition to the coaches’ views. The findings corroborate the previous relevant work and arguments pointing out the coaches’ predominant use of reproductive teaching methods during training (Cassidy et al., 2008; Ince and Kilic, 2016; Kilic and Ince, 2017). Considering the sport settings examined, the findings imply the coaches’ professional needs in providing instruction aligned to athletes’ higher-order learning needs, specifically regarding using athlete-initiated and problem-solving teaching methods. High dependence on reproductive teaching methods during training, in which various learning situations and accompanying learning needs arise, may create misalignment between the instructional methods used and the athletes’ age and competitive level contingent learning needs. The study findings on the coaches’ pervasive use of reproductive teaching methods can be associated with ‘poor coaching,’ which youth athletes from a variety of backgrounds defined as ‘not providing useful instruction, not aligning their instruction to each of athlete’s unique needs, and not being knowledgeable about the effective use of teaching methods’ (Gearity, 2012). A recent study’s findings on youth athletes’ developmental outcomes in context, which indicates a significant decrease in athletes’ developmental sport outcomes as they age (Kılıç and İnce, 2021b), may also partially give hints of ‘poor coaching’ through which youth athletes may have been excessively exposed to reproductive teaching methods regardless of their learning needs.

Coaching effectiveness is linked to coaches’ capacity to apply an array of teaching methods with the awareness of their implications on athletes’ learning (Cassidy et al., 2008, p. 40). With the precondition of having an extensive teaching method repertoire, coaches also need to have a basic understanding of the theories supporting the teaching methods and their sets of assumptions about learning when applying them (Light, 2008). While bearing structural similarities with participation context (Jones and Turner, 2006), the competitive sport may become more demanding in solving complex problems such as skill acquisition (e.g., Wikeley and Bullock, 2006). Coaches’ use of teaching methods appropriate to athletes’ cognitive, psychological, social, and emotional learning needs (Fraser-Thomas et al., 2005) may be critical for effective athlete learning (Cassidy et al., 2008). Furthermore, applying solely reproductive teaching methods in training when teaching inexperienced athletes would also be flawed as the nature of learning before formal education (sport or schools) occurs through the learner’s active engagement of discovery and problem-solving (Cassidy et al., 2008). Although important in teaching basic skills, reproductive methods are associated with behaviorist learning theory and its heavy use have been criticized for neglecting learners’ freedom, choice, and self-direction (Nelson and Colquhoun, 2013).

The descriptive analysis of coaches’ and their athletes’ responses on the value (enjoyment, learning, and motivation) given to the examined teaching methods revealed that reproductive teaching methods were ranked the highest, followed by the problem-based and athlete-initiated teaching methods. A Mann–Whitney test indicated that the athletes’ value perceptions were significantly higher than that of coaches in (1) reproductive teaching methods regarding learning and motivation; (2) problem-solving teaching methods regarding motivation; and (3) athlete-initiated teaching methods regarding enjoyment, learning, and motivation. A distinct difference between the mean scores of coaches and their athletes was observed regarding the value given to athlete-initiated teaching methods. The athletes valued athlete-initiated teaching methods significantly higher in all value components, while the coaches’ scores were comparably quite low.

Excessive reliance on using reproductive teaching methods and placing a high value on them in the study context echoes the findings of the studies conducted in the physical education context. It may be attributed to the continuation of the traditional culture associated with the sport (Cassidy et al., 2008). As a result, the athletes may also become advocates of the dominant teaching methods and follow this tradition, especially when these methods work well during the early phases of skill learning (Cassidy et al., 2008). The prevailing sport culture may impose the taken-for-granted practices involving predominantly reproductive teaching methods through interaction with other coaches or other mechanisms such as mentoring. These random learning experiences may lead to the perpetuation of existing reproductive teaching methods in coaching practices by no more than passing on ‘tricks of the trade’ (Cushion et al., 2003). On the contrary, teaching methods (A spectrum of teaching styles) were designed to enhance practitioners’ teaching approach by reflecting on their instruction (Mosston, 1972; Cassidy et al., 2008). Coaches’ reflection on their teaching experiences using problem-solving and athlete-initiated teaching methods in addition to reproductive teaching methods can help coaches evolve their instruction and, as a result, improve athletes’ holistic developmental sport outcomes (Potrac and Cassidy, 2006; Camiré et al., 2014).

While the athletes’ perceptions were found to be generally higher than that of coaches in the value given to the teaching methods examined, importantly, among other teaching methods, the athletes perceived only the athlete-initiated teaching methods as significantly more enjoyable than their coaches did. In athlete development literature, involvement in enjoyable activities is linked to young athletes’ development of intrinsic motivation through which they sustainably participate in sports and develop the resilience needed to overcome future athletic difficulties (e.g., Balyi et al., 2013, p. 52; Côté et al., 2010). According to the self-determination theory, the feeling of control over a person’s actions (autonomy) is one of the primary basic psychological needs for developing intrinsic motivation. It involves the learner’s interest, enjoyment, and inherent satisfaction (Ryan and Deci, 2017). Learner enjoyment increase in the social-contextual conditions that encourages learner autonomy in less structured activities and is critical for enhancing youth sport participation (e.g., Barnett et al., 1992; Fraser-Thomas et al., 2005).

### 4.1. Limitations

When interpreting the findings, the limitations of the study need consideration. Firstly, the study data comprised survey responses of coaches and their athletes in the competitive youth sport context. We suggest that future studies examining coaches’ instruction integrate systematic field observations and use qualitative inquiry in addition to the survey responses of coaches and their athletes to obtain a more comprehensive understanding of coaches’ capacity to apply teaching methods. Secondly, the data collection was limited to the two major cities and two little towns of Türkiye. Scanning a wider population from different coaching contexts can be done with the use of survey forms for athletes and coaches. Thirdly, in this study, the focus was on youth sports coaches and their athletes’ use and value perception of teaching methods in training without considering their age and gender subgroups in the study population. Data was collected in the natural training setting from the available population. Further studies should consider the effect of coaches’ and athletes’ age and gender on their use and value of teaching methods.

### 4.2. Conclusion and recommendations

One of the critical elements of coaching knowledge is pedagogical knowledge (Trudel et al., 2013, p. 385). Coaching effectiveness is directly affected by coaches’ capacity to use this knowledge and provide instruction tailored to athletes’ learning needs. Researchers urged a learner-centered pedagogy to athlete development to improve athletes’ higher-order thinking in the physical and psychosocial aspects of sport (e.g., Fraser-Thomas et al., 2005; Potrac and Cassidy, 2006; Wikeley and Bullock, 2006; Cassidy et al., 2008). To realize this, coaches should be well-informed about a variety of teaching methods (Cassidy et al., 2008; Pill and SueSee, 2022) and, more importantly, their theoretical roots to appropriately apply these methods according to athletes’ different learning needs (Light, 2008; Gearity, 2012; Roberts and Potrac, 2014). To develop coaches’ instructional capacities in this vein, the teaching methods coaches currently apply during their practices in a definite sport context needs careful consideration. This study provides a detailed examination of the use of and value given to reproductive, problem-solving, and athlete-initiated teaching methods in a competitive youth sport context, drawing on the tenets of the spectrum of teaching styles (Mosston and Ashworth, 2008). The study findings enabled a detailed assessment of the coaches’ pedagogical knowledge and practices from the athletes’ perspectives in addition to their perceptions of their instruction.

The study’s findings strongly indicate the coaches’ professional needs in their pedagogical knowledge, specifically their repertoire of problem-solving and athlete-initiated teaching methods and the capacity to apply them. The athletes rating athlete-initiated teaching methods as significantly more enjoyable, motivating, and instructive, contrary to the coaches, also highlights the high demand for the athlete-centered approach to instruction in the sport context studied from athletes’ part. To improve coaches’ capacity to apply knowledge in the areas the present study findings addressed, community-based learning research has been suggested in the literature (Gilbert et al., 2009; Trudel et al., 2013). Such collaborative learning environments designed based on the social theory of learning (communities of practice; Wenger, 1998) have been evidenced as effective in providing quality learning environments for coaches (e.g., Culver and Trudel, 2006; Stoszkowski and Collins, 2014; Duarte et al., 2021).

## Data availability statement

The raw data supporting the conclusions of this article will be made available by the authors, without undue reservation.

## Ethics statement

The studies involving human participants were reviewed and approved by Middle East Technical University Human Subject Ethics Committee. Written informed consent to participate in this study was provided by the participants’ legal guardian/next of kin.

## Author contributions

All authors listed have made a substantial, direct, and intellectual contribution to the work and approved it for publication.

## Conflict of interest

The authors declare that the research was conducted in the absence of any commercial or financial relationships that could be construed as a potential conflict of interest.

## Publisher’s note

All claims expressed in this article are solely those of the authors and do not necessarily represent those of their affiliated organizations, or those of the publisher, the editors and the reviewers. Any product that may be evaluated in this article, or claim that may be made by its manufacturer, is not guaranteed or endorsed by the publisher.
